# GADD45**α** is a direct target of TFEB and contributes to tacrolimus-induced chronic nephrotoxicity

**DOI:** 10.1172/jci.insight.183560

**Published:** 2025-02-06

**Authors:** Ping Gao, Xinwei Cheng, Maochang Liu, Hui Peng, Guodong Li, Tianze Shang, Jianqiao Wang, Qianyan Gao, Chenglong Zhu, Zhenpeng Qiu, Chengliang Zhang

**Affiliations:** 1 School of Pharmacy, Hubei University of Chinese Medicine, Wuhan, China.; 2 Wuhan Children’s Hospital, Tongji Medical College, and; 3 Tongji Hospital, Tongji Medical College, Huazhong University of Science and Technology, Wuhan, China.; 4 Hubei Key Laboratory of Resources and Chemistry of Chinese Medicine, Hubei University of Chinese Medicine, Wuhan, China.; 5 Hubei Shizhen Laboratory, Wuhan, China.; 6 Center of Traditional Chinese Medicine Modernization for Liver Diseases, Hubei University of Chinese Medicine, Wuhan, China.

**Keywords:** Nephrology, Therapeutics, Toxicology

## Abstract

Tacrolimus-induced chronic nephrotoxicity (TICN) hinders long-term use of tacrolimus, but its mechanism remains unclear. Tacrolimus exerts its pharmacological effect by inhibiting calcineurin and its substrate nuclear factor of activated T cells. Whether the inhibition of other calcineurin substrates is related to TICN remains to be explored. Transcription factor EB (TFEB), a substrate of calcineurin, plays a crucial role in homeostasis. Herein, we found that tacrolimus inhibited TFEB nuclear translocation and activity in mouse kidneys and HK-2 cells. Then, TFEB gain and loss of function rescued and exacerbated, respectively, the effect of tacrolimus in HK-2 cells. Furthermore, TFEB activation by both phosphorylation site mutation and agonist rescued TICN in mice. To elucidate the mechanism of TFEB, we analyzed ChIP-Seq data. We identified growth arrest and DNA damage-inducible 45α (GADD45α) as a transcriptional target of TFEB via ChIP and dual-luciferase reporter assays. Then we revealed that GADD45α overexpression rescued DNA damage and kidney injury caused by tacrolimus or TFEB knockdown in vitro and vice versa. The protective effect of GADD45α against TICN and DNA damage was further demonstrated by overexpressing it in mice. In conclusion, the persistent inhibition of the TFEB/GADD45α pathway by tacrolimus contributes to TICN. This study identifies a specific target for intervention in TICN.

## Abstract 2

The inhibition of tacrolimus on TFEB, another substrate of calcineurin, contributes to its chronic nephrotoxicity. And we identify that GADD45α is a direct target of TFEB.

## Graphical abstract

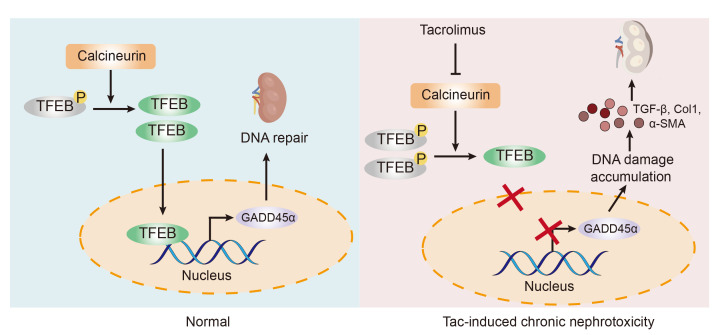

## Introduction

Tacrolimus (Tac) is the most widely used calcineurin inhibitor (CNI) in organ transplantation and is commonly used to treat autoimmune diseases (1). Despite its superior pharmacological effects, Tac was associated with multiple adverse reactions. Among them, chronic nephrotoxicity is a major obstacle that hinders the long-term clinical use of Tac. Tac-induced chronic nephrotoxicity (TICN) manifests as an irreversible progressive decline in renal function, which is an important cause of increased mortality in transplant patients (2). Its incidence ranges from 35.0% to 96.8% (3, 4). Unfortunately, there is still no effective method to prevent or alleviate TICN. To solve the dilemma, a specific target of Tac causing kidney injury must be identified.

It is well recognized that Tac exerts its immunosuppressive effect via inhibiting the phosphatase activity of calcineurin, hence hindering the dephosphorylation and translocation of nuclear factor of activated T cells (NFAT). However, the inhibition of calcineurin by Tac is not immune cell specific, and calcineurin has other substrates besides NFAT (5), both of which may result in off-target effects and even adverse reactions of Tac. Recently, Park et al. found that Tac suppressed the activation of transcription factor EB (TFEB), a substrate of calcineurin, and thus leads to β cell dysfunction and glucose intolerance, which is a common adverse reaction of Tac (6).

TFEB, a member of the microphthalmia/transcription factor E family, has been demonstrated to be a substrate of calcineurin (7, 8). In the basal state, TFEB is phosphorylated and remains inactive in the cytoplasm. Under stress, TFEB is dephosphorylated and then translocated into the nucleus, facilitating the transcription of its target genes. Calcineurin has been proven to dephosphorylate TFEB at serine 142 and serine 211, which are pivotal phosphorylation sites on TFEB (9, 10). Thus, Tac can specifically block TFEB dephosphorylation and activation by inhibiting calcineurin. TFEB can coordinate multiple signaling pathways to maintain homeostasis in an autophagy-dependent or -independent manner, so its dysfunction may result in various diseases (11, 12). It has been revealed that TFEB deficiency contributed to multiple kidney diseases, such as renal fibrosis, obesity-related kidney disease, and diabetic nephropathy (13–15). However, the relationship between Tac-suppressed TFEB and Tac nephrotoxicity remains unexplored. Therefore, the primary objective of this study was to investigate the role of TFEB in TICN.

TFEB was initially known as a pivotal regulator of lysosomes and autophagy, but an increasing body of research has confirmed its involvement in diverse fields, including DNA damage (11, 16). Accumulation of damaged DNA can trigger cell death and senescence and consequently cause loss of organ function. Therefore, DNA repair is essential to maintain cellular homeostasis. Previous studies have found that CNIs could enhance DNA damage and inhibit DNA repair (17, 18), but the mechanism has rarely been elucidated. Furthermore, whether the defective DNA repair is involved in TICN has not been studied. Therefore, the secondary purpose of the study was to investigate whether DNA damage was involved in TICN and its potential mechanism.

In the present study, we verified that Tac-blocked TFEB activation played a critical role in TICN. Moreover, we showed that DNA damage-inducible 45α (*GADD45A*), a gene responsible for DNA repair, was a direct target of TFEB and participated in TFEB inactivation–mediated TICN.

## Results

### TFEB cytosol-to-nucleus translocation is inhibited in TICN.

**Figure 1:**
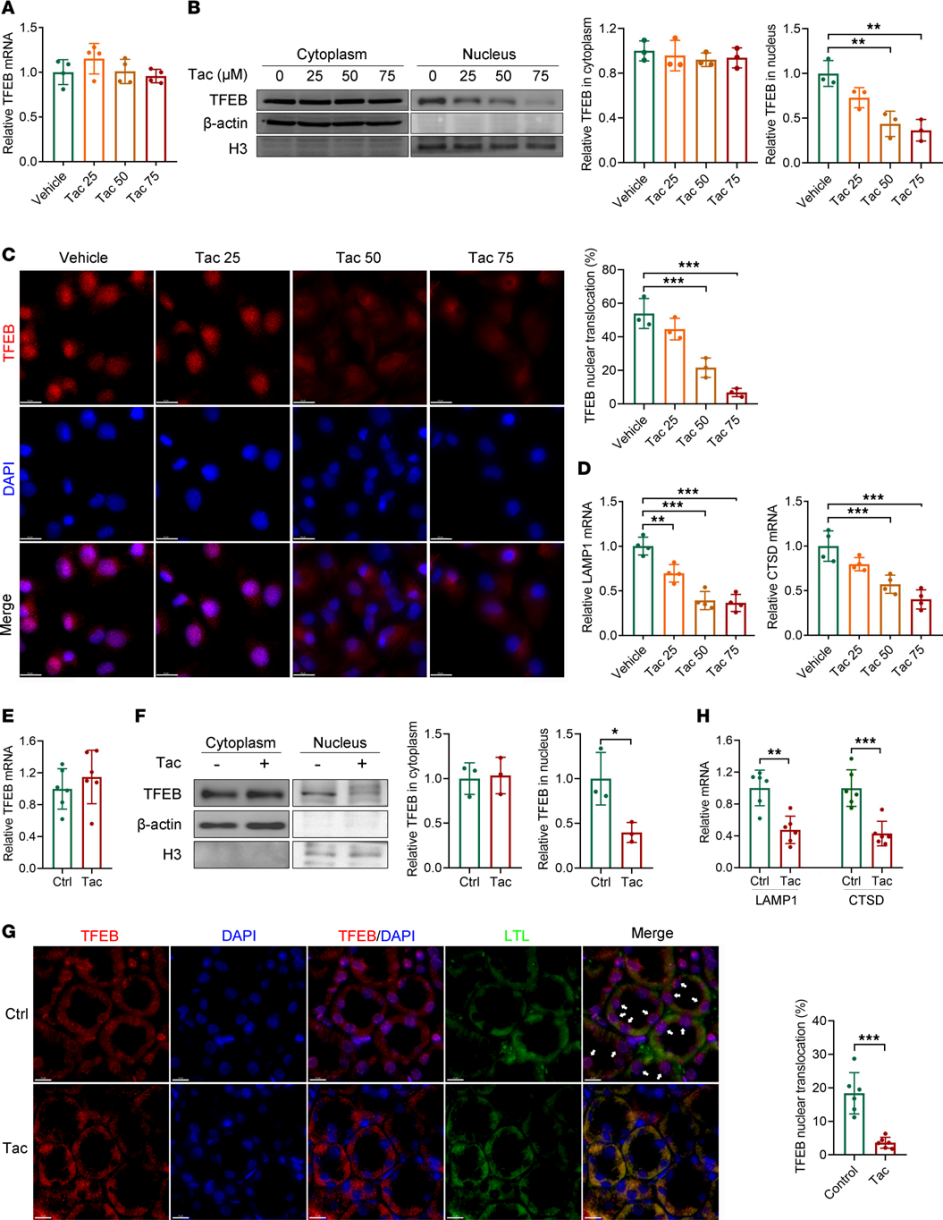
TFEB cytosol-to-nucleus translocation is inhibited in vitro and in vivo. HK-2 cells were exposed to Tac (25, 50, and 75 μM) for 24 hours, and then (**A**) *TFEB* mRNA and (**B**) protein were determined by qPCR (*n* = 4) and Western blot (*n* = 3), respectively. TFEB expression levels in cytoplasm and nucleus were normalized to β-actin and H3, respectively. qPCR, quantitative PCR. (**C**) After being starved for 4 hours, HK-2 cells were treated with vehicle or Tac for 24 hours, then measured by immunofluorescence. Scale bar, 20 μm. Nuclear TFEB fluorescence intensity was quantified (*n* = 3). (**D**) mRNA levels of *LAMP1* and *CTSD* in HK-2 cells were quantified by qPCR (*n* = 4). C57BL/6 mice were subcutaneously injected with vehicle or Tac (1.5 mg/kg/d) for 6 weeks, and then (**E**) *Tfeb* mRNA and (**F**) protein in kidney were determined by qPCR (*n* = 6) and Western blot (*n* = 3), respectively. (**G**) TFEB (shown in red) and proximal tubule marker lotus tetragonolobus lectin (LTL) (shown in green) were measured by immunofluorescence. Arrowheads indicate nuclear localized TFEB. Scale bar, 10 μm. Nuclear TFEB fluorescence intensity was quantified (*n* = 6). (**H**) mRNA levels of *Lamp1* and *Ctsd* in mouse kidneys were quantified by qPCR (*n* = 6). Data are shown as mean ± SD and analyzed by 1-way ANOVA (**A**–**D**) or 2-tailed Student’s *t* tests (**E**–**H**). *P < 0.05, **P < 0.01, ***P < 0.001.

To investigate whether TFEB is altered in TICN, we examined TFEB expression and activity in vitro and in vivo. In HK-2 cells, Tac had little effect on the *TFEB* mRNA level (Figure 1A). However, both Western blot and immunofluorescence analyses revealed that Tac inhibited TFEB nuclear translocation in a dose-dependent manner (Figure 1, B and C). The TFEB target genes lysosomal associated membrane protein 1 (*Lamp1*) and cathepsin D (*Ctsd*) were also significantly downregulated by Tac treatment in a dose-dependent manner (Figure 1D). In C57BL/6 mice, similar changes of TFEB in TICN were observed — i.e., *Tfeb* mRNA level was rarely affected (Figure 1E) — and nuclear translocation was significantly suppressed (Figure 1F). Since proximal tubules are more vulnerable to toxins, we evaluated the nuclear translocation of TFEB in proximal tubules via the costaining of the proximal tubule marker lotus tetragonolobus lectin (LTL) and TFEB (19). As shown in Figure 1G, TFEB nuclear translocation was notably weakened in proximal tubules of mice treated with Tac. mRNA levels of TFEB target genes were significantly reduced (Figure 1H). These results suggest that TFEB nuclear translocation and activity are inhibited in TICN.

### The role of TFEB in Tac-treated HK-2 cells is identified by activation and knockdown.

**Figure 2:**
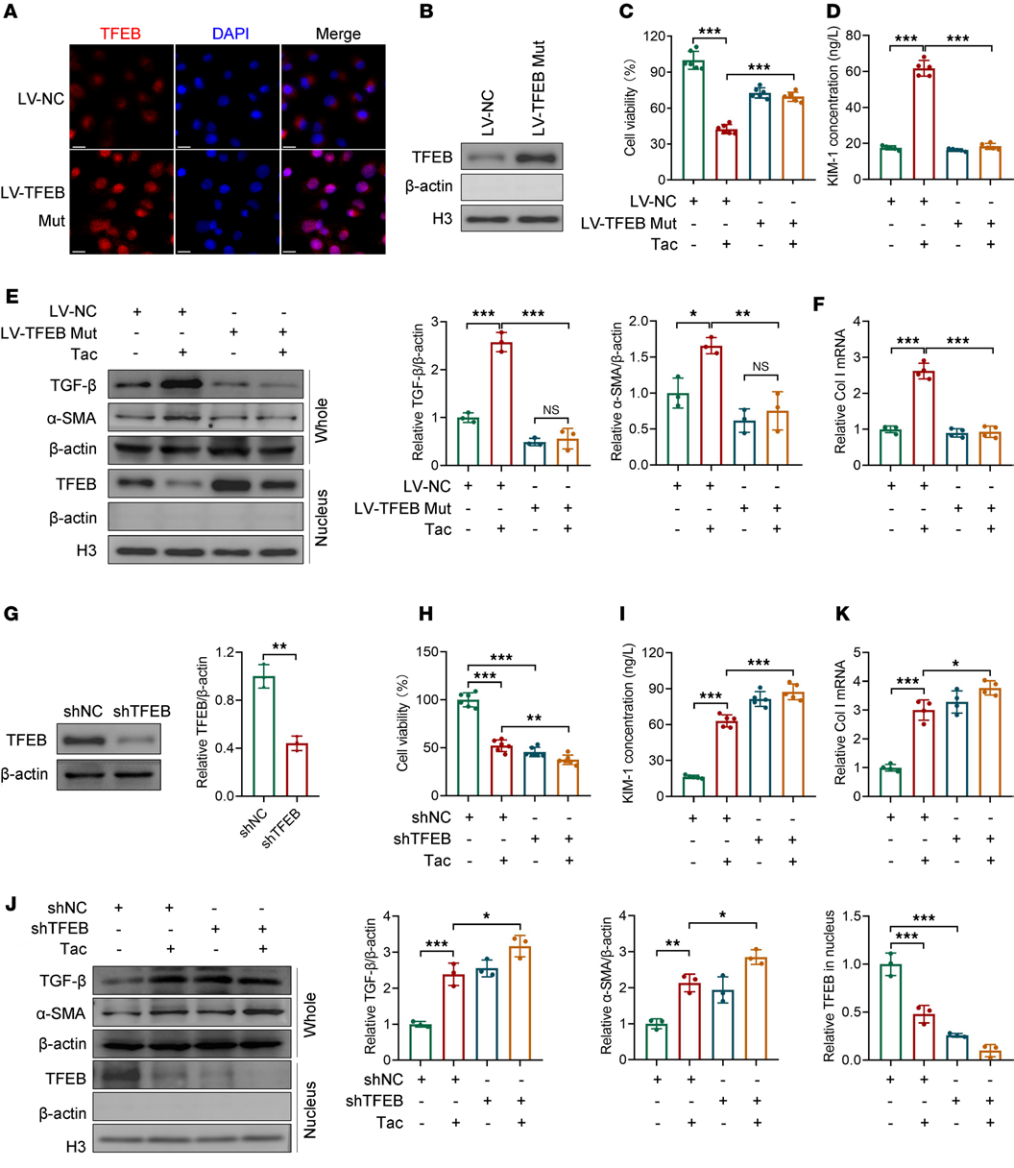
TFEB gain and loss of function modifies the effect of Tac in HK-2 cells. TFEB nuclear translocation in HK-2 cells infected with empty (LV-NC) or *TFEB*-S142A/S211A–overexpressing lentivirus (LV-*TFEB* Mut) was determined by (**A**) immunofluorescence and (**B**) Western blot (*n* = 3). Scale bar, 20 μm. (**C**) Cell viability and (**D**) KIM-1 concentration in LV-NC or LV-TFEB mutated HK-2 cells treated with vehicle or Tac (50 μM for 24 hours) were determined by CCK-8 (*n* = 6) and ELISA (*n* = 5), respectively. CCK-8, cell counting kit-8. (**E**) TGF-β and α-SMA protein levels in the whole cell and TFEB in the nucleus were determined by Western blot (*n* = 3). (**F**) *COL1A1* mRNA level was determined by qPCR (*n* = 4). (**G**) TFEB protein in HK-2 cells stably expressing control shRNA (shNC) or *TFEB* shRNA (sh*TFEB*) was determined by Western blot (*n* = 3). (**H**) Cell viability and (**I**) KIM-1 concentration in shNC or sh*TFEB* HK-2 cells treated with vehicle or Tac (50 μM for 24 hours) was determined by CCK-8 (*n* = 6) and ELISA (*n* = 5), respectively. (**J**) TGF-β and α-SMA protein levels in whole cell and TFEB in nucleus were determined by Western blot (*n* = 3). (**K**) *COL1A1* mRNA level was determined by qPCR (*n* = 4). Data are shown as mean ± SD and analyzed by 1-way ANOVA (**C**–**F** and **H**–**J**) or 2-tailed Student’s *t* tests (**G**). *P < 0.05, **P < 0.01, ***P < 0.001.

Since Tac inactivated TFEB by blocking its dephosphorylation (6, 7, 20), to rescue this effect, we constructed serine-to-alanine mutations at TFEB major phosphorylation sites 144 and 211 (S142A/S211A), then carried S142A/S211A in a lentiviral vector. Immunofluorescence and Western blot verified that S142A/S211A phospho-mutant TFEB significantly increased its nuclear translocation in HK-2 cells (Figure 2, A and B, and Supplemental Figure 1, A and B; supplemental material available online with this article; https://doi.org/10.1172/jci.insight.183560DS1). The target genes of TFEB, *LAMP1* and *CTSD*, were also increased in *TFEB*-mutated cells (Supplemental Figure 1, C and D), indicating that TFEB was successfully activated. In *TFEB*-WT cells, Tac remarkably decreased cell viability, which was significantly attenuated by expression of a constitutively active *TFEB*-mutant construct (Figure 2C and Supplemental Figure 1E). However, we found that the viability of LV-*TFEB* mutation cells was also decreased compared with control cells (*P* < 0.01), indicating that overactivation of TFEB may have adverse consequences for normal cells. Meanwhile, the kidney tubular injury marker KIM-1 was detected. As shown in Figure 2D, KIM-1 was dramatically increased by Tac in *TFEB*-WT cells, whereas the increase was abolished in TFEB-S142A/S211A cells. As for the marker of TICN, the elevation of TGF-β, α–smooth muscle actin (α-SMA), and collagen type I alpha 1 chain (*COL1A1*) by Tac was not observed in TFEB-S142A/S211A cells (Figure 2, E and F, and Supplemental Figure 1F).

To further verify the function of TFEB, we knocked down *TFEB* using shRNA, and the knockdown efficiency was determined by Western blot (Figure 2G). *TFEB* knockdown significantly impaired cell viability and increased KIM-1 level, further exacerbating Tac-induced cell damage (Figure 2, H and I). The loss of TFEB function also remarkably increased TGF-β and α-SMA protein levels and *COL1A1* mRNA (Figure 2, J and K). These results indicate that the effect of Tac in HK-2 cells is abolished by TFEB phosphorylation site mutation yet aggravated by *TFEB* knockdown in vitro.

### TICN is alleviated by activation of TFEB in vivo.

To verify whether TFEB is essential for TICN, we activated TFEB in mice using 2 methods. Torin 1, an inhibitor of mTOR, has been demonstrated to inhibit phosphorylation of TFEB (21, 22). The Western blot results suggested that Torin 1 could promote the nuclear translocation of TFEB in mouse kidney tissue (Supplemental Figure 2A). Meanwhile, Torin 1 showed little impact on kidney/body weight ratio, serum creatinine (Scr), blood urea nitrogen (BUN), renal pathology, and TICN-related markers (Supplemental Figure 2, B–H). Compared with the control group, Tac significantly reduced the mouse kidney/body weight ratio and dramatically increased the content of Scr and BUN. However, these effects of Tac were cancelled out after coadministration with Torin 1 (Supplemental Figure 2, B–D). H&E and Masson’s stainings revealed that Tac treatment led to inflammatory cell infiltration, tubular atrophy, and tubulointerstitial fibrosis, while Torin 1 significantly restored these pathological changes (Supplemental Figure 2E). Quantitative analysis of Masson’s staining demonstrated that Torin 1 significantly reduced the proportion of tubulointerstitial fibrosis caused by Tac (*P* < 0.001, Supplemental Figure 2F). In addition, Tac-induced increases of TGF-β, α-SMA, and *Col1a1* in renal tissue were notably attenuated by Torin 1 (Supplemental Figure 2, E, G, and H).

**Figure 3:**
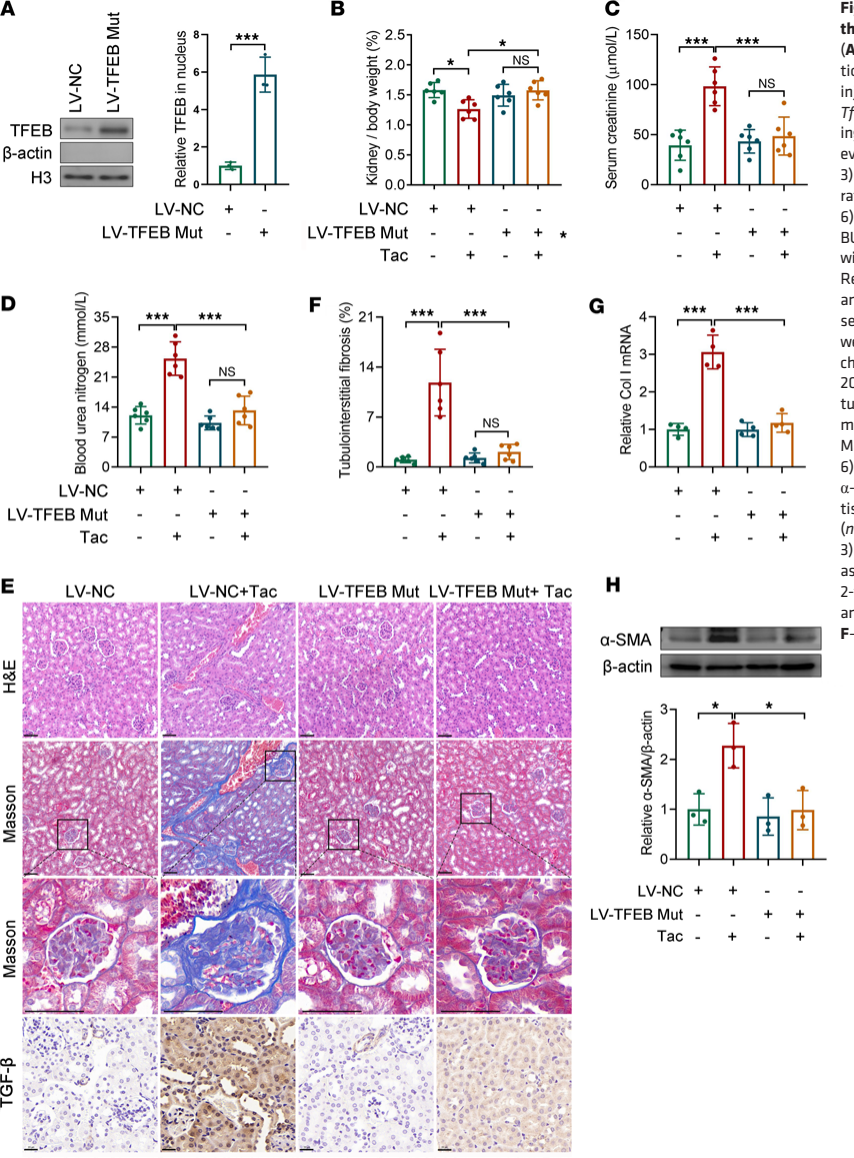
TICN is alleviated by the activation of TFEB in vivo. (**A**) TFEB nuclear translocation in kidney tissues of mice injected with empty (LV-NC) or *Tfeb*-S142A/S211A overexpressing lentivirus (LV-*Tfeb* Mut) was evaluated by Western blot (*n* = 3). (**B**) The kidney/body weight ratio of mice was recorded (*n* = 6). The levels of (**C**) Scr and (**D**) BUN in mice were determined with commercial kits (*n* = 6). (**E**) Representative images of H&E- and Masson’s-stained kidney sections (scale bar, 50 μm), as well as TGF-β immunohistochemical staining (scale bar, 20 μm). (**F**) The proportion of tubulointerstitial fibrosis in mouse renal tissue stained by Masson’s was quantified (*n* = 6). (**G**) *Col1a1* mRNA and (**H**) α-SMA protein in mouse renal tissue was measured by qPCR (*n* = 4) and Western blot (*n* = 3), respectively. Data are shown as mean ± SD and analyzed by 2-tailed Student’s *t* tests (**A**) and 1-way ANOVA (**B**–**D** and **F**–**H**). *P < 0.05, ***P < 0.001.

Since Torin 1 is an inhibitor of the mTOR pathway, it may have effects on other downstream pathways in addition to activating TFEB. Therefore, we then specifically activated TFEB via renal parenchymal injection of lentivirus carrying S142A/S211A phospho-mutant *Tfeb* in C57BL/6 mice. Compared with empty lentiviral vectors, notably increased TFEB nuclear translocations were observed in TFEB-S142A/S211A mice (Figure 3A). In wild-type mice, Tac treatment resulted in significant nephrotoxicity. However, TFEB mutation rescued TICN, manifested as remarkable improvement in kidney/body weight ratio and Scr and BUN levels in TFEB-S142A/S211A mice (Figure 3, B–D). Pathology stainings of mouse kidneys also revealed that TFEB-S142A/S211A significantly alleviated the adverse effects of Tac on the glomerulus, tubules, and interstitium (Figure 3E). Compared with control lentiviruses, TFEB variation significantly reduced the proportion of tubulointerstitial fibrosis (*P* < 0.001, Figure 3F). Besides, the elevated TGF-β, α-SMA, and *Col1a1* levels by Tac in wild-type mice were absent in TFEB-S142A/S211A mice (Figure 3, E, G, and H). Collectively, these data indicate that TICN is rescued by TFEB activation.

### GADD45α is a direct target of TFEB.

**Figure 4:**
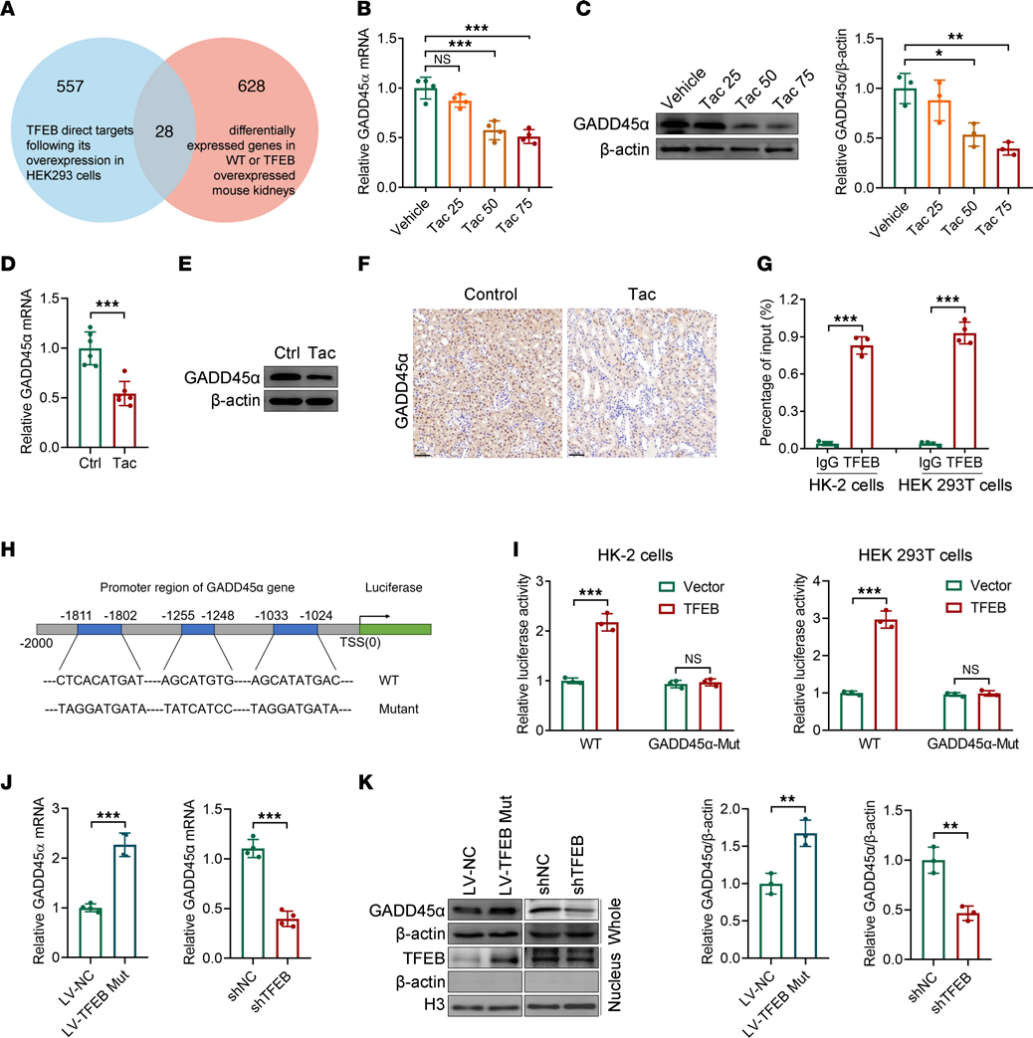
GADD45α is regulated by TFEB in TICN. (**A**) Venn diagram showing the overlap between TFEB direct target genes identified in HEK293T cells (23) and differentially expressed genes in the kidneys of wild-type or kidney-specific TFEB-overexpressed mice (24). (**B**) *GADD45A* mRNA and (**C**) protein levels in HK-2 cells treated with vehicle or Tac (25, 50, and 75 μM) for 24 hours were determined by qPCR (*n* = 4) and Western blot (*n* = 3), respectively. (**D**) *Gadd45a* mRNA and (**E** and **F**) protein in C57BL/6 mice injected with vehicle or Tac (1.5 mg/kg/d) for 6 weeks were determined by qPCR (*n* = 6), Western blot (*n* = 3), and immunohistochemistry (*n* = 6), respectively. Scale bar, 50 μm. (**G**) ChIP-qPCR was performed in HK-2 cells and HEK293T cells to identify the enrichment of TFEB onto the *GADD45A* promoter region (*n* = 4). IgG served as an antibody control. (**H**) The potential TFEB binding sites in the promoter of GADD45α and luciferase constructs were presented. (**I**) Luciferase reporter assay in HK-2 cells and HEK293T cells (*n* = 3). (**J**) *GADD45A* mRNA and (**K**) protein in HK-2 cells transfected with *TFEB*-S142A/S211A lentivirus or *TFEB* shRNA were measured by qPCR (*n* = 4) and Western blot (*n* = 3), respectively. Data are shown as mean ± SD and analyzed by 1-way ANOVA (**B** and **C**) or 2-tailed Student’s *t* tests (**D**, **G**, and **I**–**K**). *P < 0.05, **P < 0.01, ***P < 0.001.

To characterize the molecular mechanisms underlying the aggravation of TICN by TFEB inactivation, we retrieved mRNA-sequencing data from renal cells or tissues with TFEB loss or gain of function in National Center for Biotechnology Information Gene Expression Omnibus datasets. Two research studies met these criteria by December 2022. We analyzed the intersection of TFEB direct target genes identified in HEK293 cells (23) and differentially expressed genes in the kidneys of wild-type or kidney-specific *Tfeb*-overexpressed mice (24) and found 28 overlapping genes (Figure 4A and Supplemental Table 1). Among them, GADD45α plays a vital role in DNA damage repair and participates in both acute and chronic kidney injury (25, 26), so its expression in TICN was measured. In HK-2 cells, both the mRNA and protein levels of GADD45α were decreased by Tac treatment in a dose-dependent manner (Figure 4, B and C). Compared with mice given vehicle, the mRNA level of *Gadd45a* was significantly downregulated in TICN mice (Figure 4D). Western blot and immunohistochemical staining revealed that GADD45α was inhibited by Tac (Figure 4, E and F, and Supplemental Figure 3A).

Next, we tested whether *GADD45A* is a direct target gene of TFEB. ChIP-qPCR assay results indicated that TFEB could bind to the promoter of *GADD45A* both in HK-2 cells and in HEK293T cells (Figure 4G). The potential TFEB binding sites and luciferase reporter constructs are shown in Figure 4H. Dual-luciferase reporter assays exhibited that TFEB overexpression enhanced the *GADD45A* promoter-luciferase reporter more than 2-fold, whereas the elevation was abolished after mutating *GADD45A* (Figure 4I). Furthermore, we verified the expression of GADD45α in TFEB-S142A/S211A and TFEB-knockdown cells. Compared with their respective empty vectors, TFEB-S142A/S211A promoted higher mRNA levels of *GADD45A* while TFEB knockdown lowered *GADD45A* mRNA (Figure 4J). Consistent with the qPCR results, the protein level of GADD45α was elevated by TFEB-S142A/S211A and decreased by TFEB knockdown (Figure 4K and Supplemental Figure 3, B and C). These results demonstrated that GADD45α is a direct transcriptional target of TFEB.

### GADD45α mediates the renal protective and DNA repair effects of TFEB in vitro.

**Figure 5:**
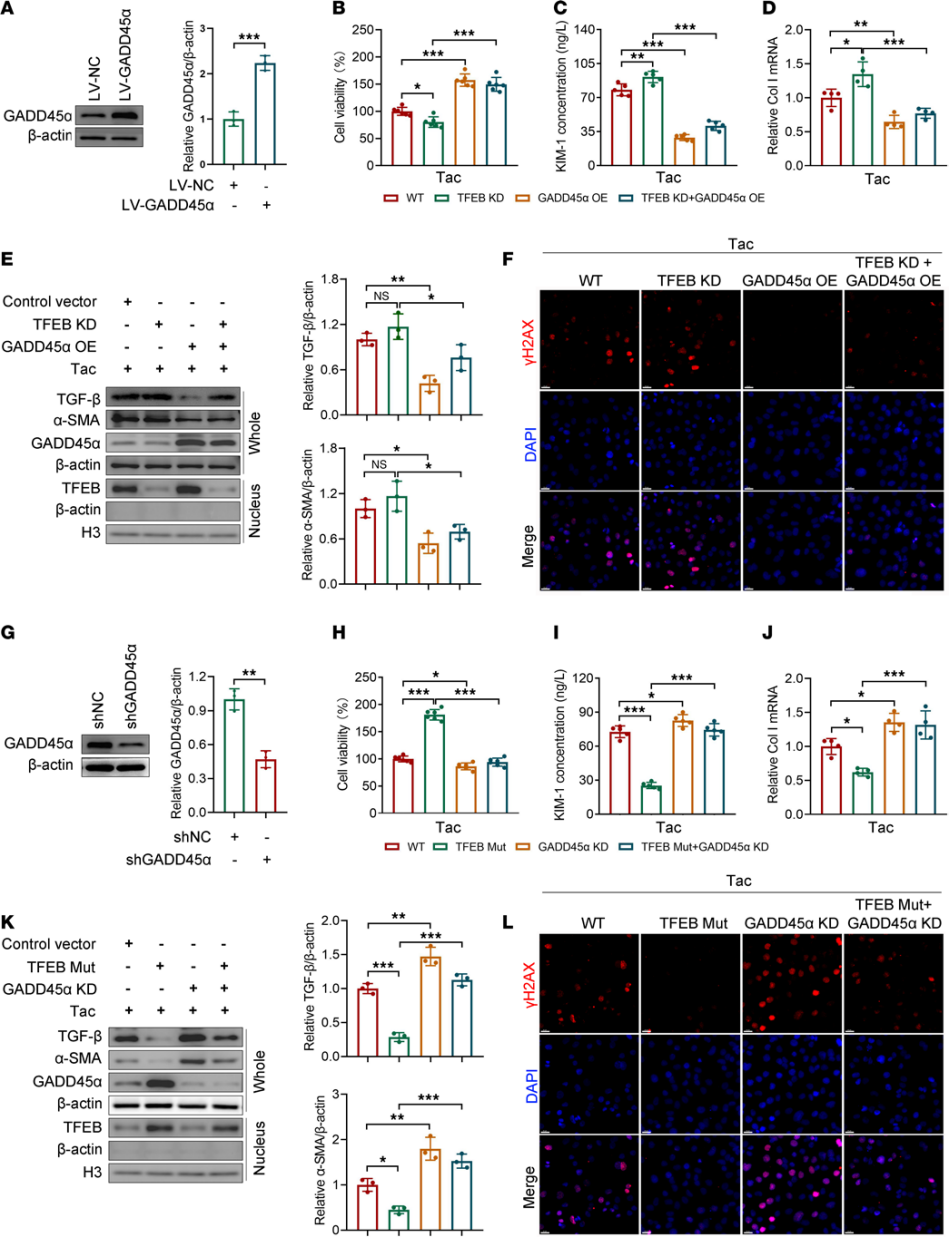
GADD45α mediates the role of TFEB in Tac-treated HK-2 cells. (**A**) The GADD45α protein level in HK-2 cells infected with empty (LV-NC) or *GADD45A* overexpression lentivirus (LV-GADD45α) was determined by Western blot (*n* = 3). (**B**) Cell viability, (**C**) KIM-1 concentration, (**D**) *COL1A1*, and (**E**) TGF-β, α-SMA, and GADD45α protein in the whole cell, as well as TFEB in the nucleus. (**F**) γH2AX levels in HK-2 cells transfected with control vectors, *TFEB* shRNA, LV-*GADD45A*, or combination of the latter 2 and then treated with Tac (50 μM for 24 hours) were determined by CCK-8 (*n* = 6), ELISA (*n* = 5), qPCR (*n* = 4), Western blot (*n* = 3), and immunofluorescence (*n* = 4). Scale bar, 20 μm. (**G**) GADD45α protein in HK-2 cells transfected with control (shNC) or *GADD45A* shRNAs (sh*GADD45A*) was determined by Western blot (*n* = 3). (**H**) Cell viability, (**I**) KIM-1 concentration, (**J**) *COL1A1*, and (**K**) TGF-β, α-SMA, and GADD45α protein in the whole cell, as well as TFEB in the nucleus. (**L**) γH2AX levels in HK-2 cells transfected with control vectors, LV-*TFEB* Mut, sh*GADD45A*, or combination of the latter 2 and then treated with Tac (50 μM for 24 hours) were determined by CCK-8 (*n* = 6), ELISA (*n* = 5), qPCR (*n* = 4), Western blot (*n* = 3), and immunofluorescence (*n* = 4). Scale bar, 20 μm. Data are shown as mean ± SD and analyzed by 2-tailed Student’s *t* tests (**A** and **G**) and 1-way ANOVA (**B**–**E** and **H**–**K**). *P < 0.05, **P < 0.01, ***P < 0.001. KD, knockdown; OE, overexpression.

Given that Tac inhibited the TFEB/GADD45α pathway, we then explored whether GADD45α overexpression could rescue the effects of Tac and TFEB knockdown in HK-2 cells. The expression of GADD45α in HK-2 cells transfected with *GADD45A* overexpression lentivirus was verified by Western blot (Figure 5A). Compared with the empty vector, *GADD45A* overexpression remarkably reversed Tac-impaired cell viability (Figure 5B), alleviated Tac-induced renal tubule damage (Figure 5C), and restored the levels of *COL1A1*, TGF-β, and α-SMA (Figure 5, D and E, and Supplemental Figure 4, A and B). Moreover, the nephrotoxic effects of Tac in *TFEB*-knockdown cells were abolished by *GADD45A* overexpression (Figure 5, B–E). Last, we measured the level of γH2AX, a marker of DNA damage. Immunofluorescence results showed that the elevated γH2AX expression by Tac treatment in wild-type and *TFEB*-knockdown cells was substantially relieved in *GADD45A*-overexpressing cells (Figure 5F).

To further examine the role of GADD45α in TFEB-mediated TICN, *GADD45A*-knockdown or empty plasmid was transfected into wild-type and TFEB-S142A/S211A cells. The knockdown of *GADD45A* was verified by Western blot (Figure 5G). Consistent with the above results, the effect of Tac in HK-2 cells was reversed by *TFEB* mutation (Figure 5, H–K). However, *GADD45A* knockdown abolished the protective effect of TFEB activation, as evidenced by notably decreased cell viability and elevated KIM-1, *COL1A1*, TGF-β, and α-SMA (Figure 5, H–K, and Supplemental Figure 4, C and D). In addition, the expression of γH2AX was reduced by TFEB activation but reversed by *GADD45A* knockdown (Figure 5L). Taken together, these data indicate that TFEB depends on GADD45α to alleviate Tac-induced renal tubular cell injury and DNA damage.

### GADD45α overexpression rescues DNA damage and TICN in vivo.

**Figure 6:**
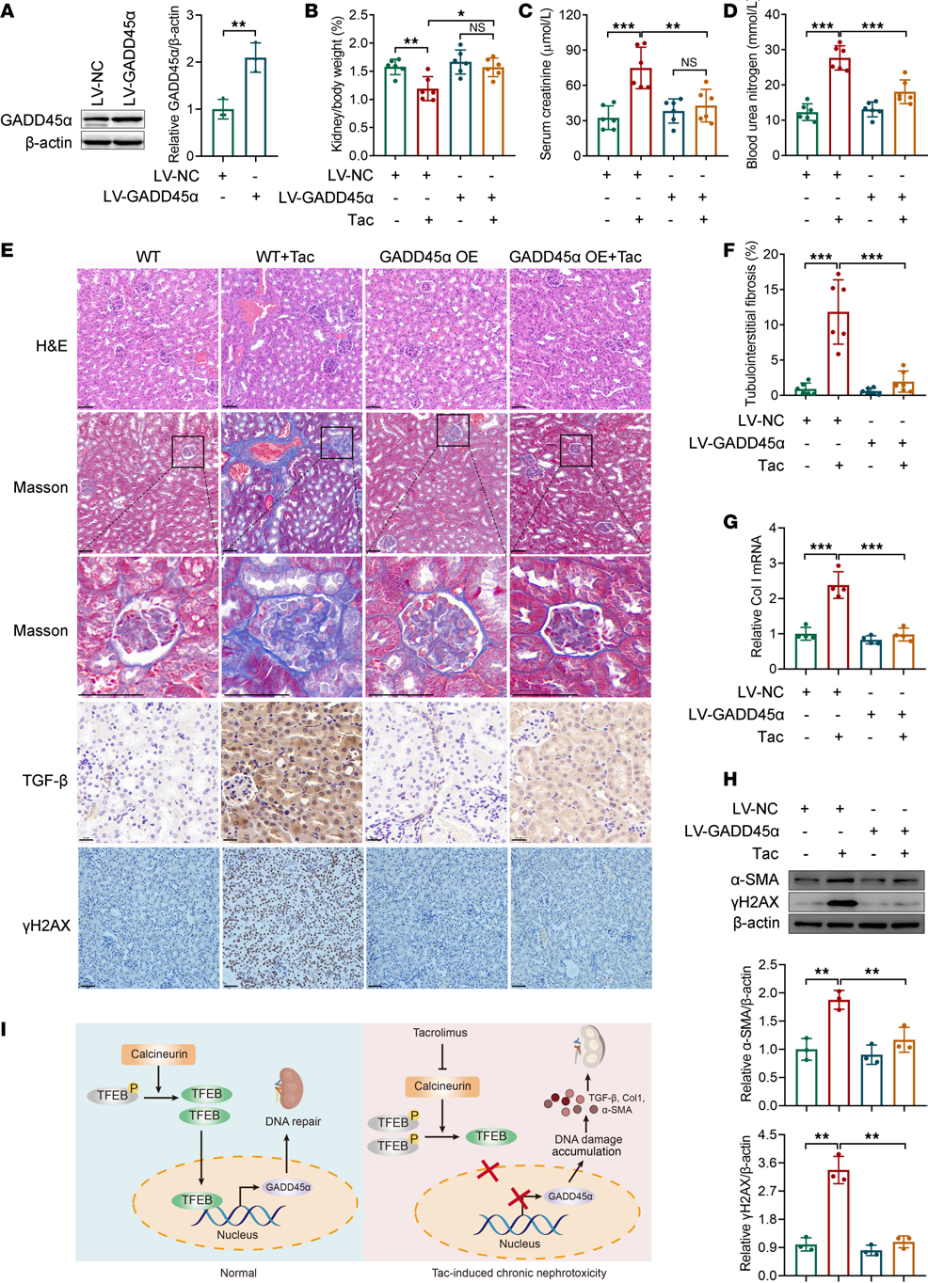
Overexpression of GADD45α reduces DNA damage and TICN in vivo. (**A**) GADD45α protein in kidney tissues of mice injected with empty (LV-NC) or *Gadd45a*-overexpressing lentivirus (LV-*Gadd45a*) was measured by Western blot (*n* = 3). (**B**) The kidney/body weight ratio of mice was recorded (*n* = 6). (**C** and **D**) The levels of Scr and BUN in mice were determined with commercial kits (*n* = 6). (**E**) Representative images of H&E- and Masson’s-stained kidney sections (scale bar, 50 μm), as well as TGF-β (scale bar, 20 μm) and γH2AX immunohistochemical staining (scale bar, 50 μm). (**F**) The proportion of tubulointerstitial fibrosis in mouse renal tissue stained by Masson’s was quantified (*n* = 6). (**G**) *Col1a1* mRNA and (**H**) α-SMA and γH2AX protein levels in mouse renal tissue were measured by qPCR (*n* = 4) and Western blot, respectively (*n* = 3). (**I**) Proposed model for the mechanism underlying TICN. Data are shown as mean ± SD and analyzed by 2-tailed Student’s *t* tests (**A**) and 1-way ANOVA (**B**–**D** and **F**–**H**). *P < 0.05, **P < 0.01, ***P < 0.001.

Finally, we verified whether overexpression of GADD45α could mitigate TICN and DNA damage in vivo. The efficiency of *Gadd45a* overexpression in mouse kidney was confirmed by Western blot (Figure 6A). Compared with wild-type mice, *Gadd45a* overexpression protected mice from Tac-induced kidney/body weight ratio loss, as well as elevation of Scr and BUN levels (Figure 6, B–D). H&E and Masson’s stainings of kidney tissues exhibited that the renal damage caused by Tac was reversed by *Gadd45a* overexpression (Figure 6E). The quantitative analysis of Masson’s-stained tissues also revealed that the proportion of Tac-induced tubulointerstitial fibrosis was significantly reduced by *Gadd45a* overexpression (*P* < 0.001) (Figure 6F). Moreover, Tac failed to increase the expression of *Col1a1*, TGF-β, and α-SMA in *GADD45a*-overexpressed mice (Figure 6, E, G, and H). As shown in Figure 6, E and H, Tac significantly increased the expression of γH2AX in the kidneys of wild-type mice, whereas this effect was mitigated by *Gadd45a* overexpression. Together, these in vivo results reaffirm that GADD45α is essential in Tac-induced nephrotoxicity and DNA damage.

## Discussion

Irreversible chronic nephrotoxicity has plagued the clinical use of Tac, but so far there is still no good relief method. One reason is the lack of specific targets for TICN. This study validated the critical role of TFEB, one of the substrates of calcineurin, in TICN, which provided a specific candidate target for the prevention of TICN. In addition, we demonstrated that GADD45α was a direct target gene of TFEB, and it played an essential role in TFEB dysfunction–mediated TICN and DNA damage.

Inhibition of calcineurin is a necessary pathway for Tac to exert its pharmacological effects. Nevertheless, the sword is double-edged. The nonspecific distribution and multisubstrate properties of calcineurin may pose potential risks for its inhibitors (5, 6, 27). In this study, we explored the role of TFEB, a substrate of calcineurin, in TICN. First, we found that Tac remarkably reduced TFEB nuclear translocation and transcriptional activity in renal tissue and tubular cells in a dose-dependent manner (Figure 1). As a recognized substrate of calcineurin, TFEB has been reported to be inactivated by Tac in other tissues (6, 20, 28). Subsequently, we verified the role of TFEB in TICN through gain- and loss-of-function methods. Considering that Tac inhibits TFEB activation by suppressing its dephosphorylation, simple overexpression of TFEB may result in the majority of TFEB being degraded by phosphorylation. Therefore, with reference to previous literature (9, 26, 29), we constructed a variant with TFEB phosphorylation site mutation, then overexpressed it with lentivirus. The experimental results revealed that TFEB-S142A/S211A did significantly increase TFEB nuclear translocation (Figure 2, A and B, and Figure 3A). By using TFEB mutation and knockdown, as well as its activator Torin 1, we demonstrated the critical role of TFEB in TICN in vivo and in vitro (Figures 2 and 3).

These data provided substantial evidence for the association between TFEB inactivation and TICN. Meanwhile, we found that TFEB nuclear translocation and GADD45α expression levels were also inhibited in Tac-induced acute kidney injury mice (data not shown), but the role of the TFEB/GADD45α pathway in Tac-induced acute nephrotoxicity needs to be further verified. Given the wide distribution of calcineurin and TFEB in the human body, the inhibition of TFEB by Tac may not be solely associated with its nephrotoxicity. Since TFEB coordinates various physiological processes, its dysfunction is also implicated in multiple diseases (11, 30, 31). TFEB has been found to be associated with Tac-induced β cell dysfunction and glucose intolerance (6). Whether it plays roles in other Tac-related adverse reactions remains to be studied. If this hypothesis is true, TFEB may be the most promising target to rescue multiple adverse reactions of Tac.

TFEB is involved in various pathways and functions, including autophagy-dependent and -independent functions (11). To uncover the target gene for TFEB, we analyzed published mRNA-sequencing results and identified 28 potential target genes. Among them, GADD45α has been demonstrated to inhibit tissue fibrosis (32, 33), an essential pathological manifestation of TICN. Hence GADD45α was tested. First, we found that GADD45α was inhibited in Tac-treated cells and TICN mice. Then, ChIP-qPCR and dual-luciferase reporter assays showed that GADD45α was the direct transcription target of TFEB (Figure 4). Meanwhile, it was upregulated in TFEB-activated cells, and vice versa, downregulated in TFEB-knockdown cells. Furthermore, GADD45α overexpression and knockdown could rescue the impacts of TFEB inactivation and activation, respectively. These data verify that GADD45α is a direct transcription target of TFEB and mediates the renal protective effect of TFEB in TICN.

GADD45α plays a critical role in DNA repair and demethylation as well as cell cycle arrest and cell senescence. TFEB is also involved in DNA repair. Previous research suggested that TFEB was activated under exposure to DNA-damaging drugs and that its knockout led to dysregulation of the DNA damage response (16, 34). Meanwhile, faulty DNA repair results in the development of acute and chronic kidney injury, as well as drug-associated nephrotoxicity (9). The most well-known drugs that cause DNA damage are chemotherapeutic drugs, particularly platinum-based agents. In fact, other drugs, such as aristolochic acid, can also cause DNA damage and hence impair kidneys (9). Although it has long been reported, DNA damage caused by CNIs has received little attention (17). As shown in Figure 6H, long-term Tac treatment did lead to significant DNA damage. Moreover, GADD45α-mediated DNA repair could mitigate TICN, as evidenced by GADD45α gain- and loss-of-function experiments. That is, Tac can impair renal DNA repair function by reducing GADD45α expression. Therefore, when the kidney is faced with persistent Tac stimulation, it fails to recover from the injury, hence causing cell cycle arrest, atrophy, and even fibrosis, driving the TICN progression.

In summary, we demonstrate that Tac blocking of TFEB dephosphorylation is responsible for its chronic nephrotoxicity. Moreover, our results verify that GADD45α is a direct transcription target of TFEB, and the loss of GADD45α-mediated DNA repair contributes to TICN (Figure 6I). Given that no effective treatment for TICN has been recognized so far, this study provides a potential specific target for the prevention and treatment of TICN. In addition, since calcineurin is the common pharmacological target for all CNIs, whether persistent suppression of the calcineurin/TFEB/GADD45α pathway plays a role in the nephrotoxicity of other CNIs, i.e., cyclosporin A, needs to be further investigated.

## Methods

### Sex as a biological variable.

Only male animals were used in this study because the incidence of TICN appears to be higher in male humans than in females (35). We expect our findings to be relevant to both male and female individuals.

### Cell culture and treatment.

Cell culture and treatment protocols were consistent with what we have previously reported (36). The cells were sourced from Shanghai Zhong Qiao Xin Zhou Biotechnology Co., Ltd. (China). Human kidney proximal tubular epithelial cells (HK-2) were grown in DMEM/F12 (Gibco) enriched with 10% FBS (Gibco) and a 1% blend of penicillin and streptomycin antibiotics. HEK293T cells underwent cultivation in DMEM (Gibco) enriched with 10% FBS and 1% penicillin/streptomycin antibiotic mixture. The cells were incubated at a temperature of 37°C within a moisture-controlled setting composed of 5% CO_2_ and 95% O_2_. Following 3–5 cycles, the cells were separated using 0.25% trypsin (GENOM) and then seeded onto collagen-layered, 6-well plates at a density of 0.5 × 10^5^ to 1 × 10^5^. For dose-response studies, cells were treated with 25, 50, and 75 μM Tac for 24 hours and other studies with 50 μM Tac. When using immunofluorescence to determine the effect of Tac dose on the subcellular localization of TFEB, HK-2 cells were starved for 4 hours before treatments were initiated.

### Animal experiments.

Male C57BL/6 mice, aged 8 weeks, were purchased from GemPharmatech (China). The mice had unrestricted access to water and were accommodated in a 12-hour light/12-hour dark cycle. After acclimation for 1 week, each mouse received a diet low in sodium (0.01% sodium) for an additional week before starting treatment. This diet was maintained throughout the entire duration of the experiment. Lentivirus-mediated gene transfer was conducted in kidneys through intraparenchymal injection at a dosage of 5 × 10^7^ transducing units/mouse. Treatment was given 1 week after the injection. As we have reported previously (36), mice in the control group received subcutaneous administration of 10 mL/kg/d olive oil (Aladdin Reagent Co., Ltd.). Mice in the model group received a subcutaneous injection of 1.5 mg/kg/d Tac (T101160, purity ≥ 98%, Aladdin Reagent Co., Ltd.) for 6 weeks. Besides Tac, mice in Torin 1 groups received simultaneously Torin 1 at 5 mg/kg/d (T129642, purity ≥ 97%, Aladdin Reagent Co., Ltd.) by intraperitoneal administration for 6 weeks. Twenty-four hours after the last dose, the mice were anesthetized and sacrificed, and their bodies and kidneys were weighed. Blood and kidneys were collected for further use. All the mice were bred and kept in environments free of pathogens.

### qPCR.

Total RNA in HK-2 cells or mouse kidney tissues was extracted with TransZol reagent (TransGen). Each sample’s total RNA (1 μg) underwent reverse transcription into cDNA via a PrimeScript RT Master Mix (Takara Shuzo) adhering to the guidelines provided by the manufacturer. Subsequently, 1 μL of the produced cDNA was used for PCR.

The following primers were employed: human *TFEB* primers (forward: 5′-TGTTGCTGCATGCGCTC-3′; reverse: 5′-CGGCAGTGCCTGGTACAT-3′), mouse *Tfeb* primers (forward: 5′-AACAAAGGCACCATCCTCAA-3′; reverse: 5′-GGAGCCAGAGCTGCTTGTTA-3′), human *LAMP1* primers (forward: 5′-CACGAGAAATGCAACACGTTA-3′; reverse: 5′-CTGGGTGCCACTAACACATCT-3′), mouse *Lamp1* primers (forward: 5′-ACCTGTCGAGTGGCAACTTCA-3′; reverse: 5′-GGGCACAAGTGGTGGTGAG-3′), human *CTSD* primers (forward: 5′-GCAAACTGCTGGACATCGCTTG-3′; reverse: 5′-GCCATAGTGGATGTCAAACGAGG-3′), mouse *Ctsd* primers (forward: 5′-TAAGACCACGGAGCCAGTGTCA-3′; reverse: 5′-CCACAGGTTAGAGGAGCCAGTA-3′), human *GADD45A* primers (forward: 5′-TTGCAATATGACTTTGGAGGAA-3′; reverse: 5′-CATCCCCCACCTTATCCAT-3′), mouse *Gadd45a* primers (forward: 5′-AGAAGACCGAAAGGATGGAC-3′; reverse: 5′-CACGGATGAGGGTGAAATG-3′), and *Gapdh* primers (forward: 5′-CAAGGTCATCCATGACAACTTTG-3′; reverse: 5′-GTCCACCACCCTGTTGCTGTAG-3′).

PCR assays were conducted on a QuantStudio 7 Flex Real-Time PCR system (Applied Biosystems) using SYBR Green Master Mix (Applied Biosystems). Comparative Ct (2^-ΔΔCt^) method was applied to analyze the quantification of mRNA expression of genes. *Gapdh* mRNA served as an internal standard to ensure accurate normalization of quantitative data.

### Protein extraction and Western blotting.

Total protein was extracted from cells and kidneys using RIPA lysis, which involves inhibitors of proteinase and phosphatase (Servicebio Technologies, Inc.). The isolation of nuclear and cytosolic segments was conducted with a Nuclear-Cytosol Extraction Kit (Applygen Technologies Inc), adhering to the guidelines provided by the manufacturer. The procedures for Western blot analysis adhered to the protocols we had previously set up (36). Antibodies for TFEB (no. 83010), Col1A1 (no. 91144), GADD45α (no. 4623S), and phospho-histone H2AX (no. 9718T) were supplied by Cell Signaling Technology. Anti–TGF-β (sc-130348) was supplied by Santa Cruz Biotechnology. β-Actin (ab8226) and secondary antibodies (ab205718, ab205719) were provided by Abcam. Images were taken using Micro Chemi (DNR Bio-imaging systems), and NIH ImageJ software quantified the observed bands. We used β-actin as the standard for loading. Each assay was conducted a minimum of 3 times.

### Immunofluorescence.

Sections of mouse kidneys, embedded in paraffin, underwent deparaffinization for later antigen recovery. Subsequently, the samples were treated with 10% goat serum in PBS for 30 minutes and then subjected to an overnight incubation at 4°C with a primary antibody. Antibodies for TFEB (no. 83010) and γH2AX (no. 9718T) were purchased from Cell Signaling Technology. After 3 consecutive 5-minute washes in PBS, primary antibodies were detected using the CY3-labeled (HY-P81017) or Alexa Fluor 488–labeled (HY-P8002) secondary antibodies purchased from MedChemExpress for 50 minutes, followed by a 10-minute application of the fluorescent nuclear DAPI stain at room temperature.

HK-2 cells were initially placed on coverslips and subsequently stabilized using 4% paraformaldehyde for a duration of 20 minutes. Subsequently, they underwent permeabilization using 0.2% Triton X-100 (Kerui) and were blocked with 1% BSA. Cells underwent an overnight incubation at 4°C with various primary antibodies. Subsequently, the process involved staining with secondary antibodies at ambient temperature for another hour, followed by a 5-minute application of the fluorescent nuclear DAPI stain. Fluorescent images were captured using a Nikon Eclipse C1 fluorescence microscope with Nikon DS-U3 camera.

In cell and animal experiments, 3 and 6 biological replicates were performed for immunofluorescence staining in each group, respectively. Also, 5 independent fields in each replicate were randomly selected for quantitative analysis. In each field, the fluorescence intensities of TFEB in nucleus and cytoplasm were measured separately. The TFEB nuclear translocation ratio is equal to the fluorescence intensity of TFEB in the nucleus divided by the total TFEB fluorescence intensity. The average of these ratios at 5 fields is regarded as the proportion of TFEB nuclear translocation for that group.

### Cell transfection.

The phosphorylation site–defective *TFEB*-mutant (S142A/S211A) and *GADD45A* overexpression lentiviruses were purchased from Gene Chem Co., Ltd. The cells were cultured until they attained a 50% confluence level in 6-well plates. Next, the *TFEB*-mutant (S142A/S211A) and *GADD45A* overexpression and corresponding negative control (NC) lentiviruses were added to the cultures for 12 hours (MOI = 5) with HitranG A (Gene Chem Co., Ltd.). The TFEB and GADD45α shRNA plasmid (including an NC) were obtained from Gene Chem Co., Ltd. The application of the E-trans transfection agent (Gene Chem Co., Ltd.) followed the guidelines provided by the manufacturer. Following a 48-hour period of disruption, the effectiveness of protein knockdown was examined using Western blot.

### Cell viability assay.

The survival of cells was evaluated with the aid of CCK-8 (Servicebio Technologies, Inc.). HK-2 cells, planted in 96-well plates, were grown in DMEM/F12 medium after a 24-hour exposure to either vehicle or Tac. Each well received a CCK-8 solution and was incubated for an hour, and then absorbance was measured at 450 nm. The findings were standardized in terms of percentage when compared with the control group.

### ELISA.

Supernatant samples from HK2 cells treated with Tac were collected. Subsequently, the concentration of KIM-1 in the cell’s supernatant was measured using the guidelines provided in the ELISA kit (Coibo Biotechnology Co., LTD).

### Kidney histology.

Following a rinse with PBS solution, the kidney tissues were preserved in 10% buffered formaldehyde and encased in paraffin. To evaluate kidney disease and fibrosis, staining with H&E and Masson’s trichrome was performed. Tubulointerstitial fibrosis is characterized by the enlargement of the interstitial space rich in matrix, along with tubular enlargement, tubular shrinkage, creation of tubular casts, removal of tubular epithelial cells, or increased thickness of the tubular basement membrane in kidney sections stained with Masson’s trichrome (37). Using Image-Pro Plus software (Media Cybernetics), the extent of fibrosis was assessed based on the proportion of damaged area in each field across at least 10 fields per section. A pathologist unaware of the treatment categories conducted histopathological examinations on randomly chosen cortical areas of kidney slices.

### Biochemical assay.

The concentrations of Scr and BUN in C57BL/6 mice were measured with commercial kits (Jiancheng Bioengineering Institute).

### Immunohistochemistry.

Kidney specimens were preserved using formalin and encased in paraffin for regular sectioning. For immunohistochemistry, sections were placed sequentially into the xylene, absolute ethanol, 85% ethanol, 75% ethanol, and distilled water to dewax and rehydrate. Then the tissue sections were placed in citrate antigen retrieval buffer (pH 6.0), and we performed the antigen retrieval in a microwave oven. After natural cooling, the sections were placed in PBS (pH 7.4) and washed on a shaker 3 times. Unspecific binding sites were blocked with 5% BSA in PBS for 30 minutes at room temperature followed by incubation with anti–TGF-β (Santa Cruz Biotechnology, sc-130348), anti-GADD45α (Cell Signaling Technology, 4632S), or anti-γH2AX (Cell Signaling Technology, 9718T) overnight at 4°C. Then they were incubated with the appropriate secondary antibodies for 50 minutes at room temperature.

### ChIP-qPCR assay.

HK-2 cells underwent fixation in formaldehyde for a duration of 10 minutes to trigger the cross-linking of DNA and proteins. Subsequently, ultrasound technology was employed to fragment the chromatin. Following the cells’ centrifugation at 12,000*g* and 4°C for a duration of 10 minutes, the supernatant obtained was gathered and split into a pair of tubes. Subsequently, the standard mouse IgG antibody (Abcam, ab172730) served as an NC, and the specific antibody targeting protein TFEB (Cell Signaling Technology, 83010) was introduced, each followed by an overnight incubation at 4°C. For the precipitation of DNA-protein complexes, a combination of protein agarose and sepharose was employed, succeeded by a 5-minute centrifugation at 12,000*g* and subsequent supernatant removal. Nonspecific compounds were washed away and de–cross-linked overnight at 65°C. Subsequently, phenol/chloroform extraction method was employed to extract and purify the DNA fragments, which were then analyzed using qPCR with GADD45α primer.

### Dual-luciferase reporter assays.

Analysis of TFEB’s binding sites on GADD45α’s promoter region was conducted using the JASPAR database (https://jaspar.elixir.no/). Putative binding sites were filtered using a significance threshold set at 0.8. The GADD45α promoter or its mutagen was subcloned into the pGL3 Basic vector (Gene Chem Co., Ltd.), followed by transfection into HK-2 cells with Lipofectamine 2000 (Invitrogen) for control cells or HK-2 cells overexpressing TFEB, as per the manufacturer’s instructions. Following a 24-hour transfection period, the Dual-Luciferase Reporter Assay system (Promega) was employed to detect luminescence. The derived figures were adjusted in relation to the activity of Renilla luciferase.

### Statistics.

All data were presented as the means ± SDs. The differences between the 2 groups were analyzed using 2-tailed Student’s *t* tests. Comparisons among various groups were conducted through a 1-way ANOVA, succeeded by Tukey’s post hoc analysis. Prism software (GraphPad Prism 7) was utilized for analyzing data and creating graphical depictions. A *P* value less than 0.05 was deemed to hold statistical significance.

### Study approval.

All animal experiments were approved by Tongji Hospital, Tongji Medical College, Huazhong University of Science and Technology (Wuhan, China).

### Data availability.

Values for all data points in graphs are reported in the Supporting Data Values file.

## Author contributions

PG, XC, ZQ, and C Zhang designed the research; PG, XC, ML, HP, GL, TS, JW, QG, and C Zhu performed the main experiments; PG and XC analyzed the data; PG and XC wrote the paper; ML, HP, GL, TS, JW, QG, C Zhu, ZQ, and C Zhang revised the paper; and ZQ and C Zhang supervised the study.

## Supplementary Material

Supplemental data

Unedited blot and gel images

Supporting data values

## Version 1 02/06/2025

In-Press Preview

## Version 2 03/24/2025

Electronic publication
