# Supplementary material for: GADD45α is a direct target of TFEB and contributes to tacrolimus-induced chronic nephrotoxicity
Source: JCI Insight. 2025 Feb 6;10(6):e183560. doi: 10.1172/jci.insight.183560 (PMC11949043; doi:10.1172/jci.insight.183560)
Supplement: Supplemental data [file jciinsight-10-183560-s044.pdf]

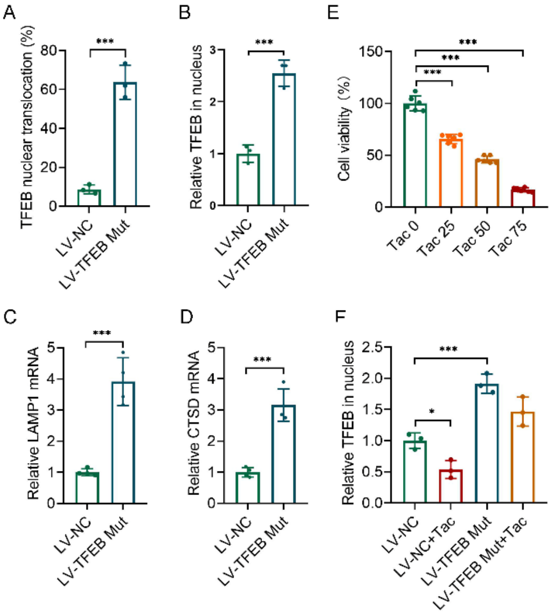

Supplemental Figure 1. (**A**) TFEB fluorescence intensity in HK-2 cells infected with empty (LV-NC) or *TFEB*-S142A/S211A overexpressing lentivirus (LV-*TFEB* Mut) was quantified (n=3). (**B**) TFEB expression levels in HK-2 cells infected with LV-NC or LV-*TFEB* Mut were normalized to H3 (n=3). (**C, D**) *LAMP1* and *CTSD* mRNA levels in LV-NC and LV-*TFEB* Mut cells were determined by qPCR (n=3). (**E**) Cell viability of HK-2 cells treated with different concentrations of tacrolimus (25, 50, 75 μM). (**F**) TFEB expression levels in HK-2 cells infected with LV-NC or LV-*TFEB* Mut treated with or without tacrolimus were normalized to H3 (n=3). Data are shown as mean ± SD and analyzed by two-tailed Student's t-tests (A-D) and one-way ANOVA (E and F). *$P < 0.05$, **$P < 0.01$, ***$P < 0.001$.

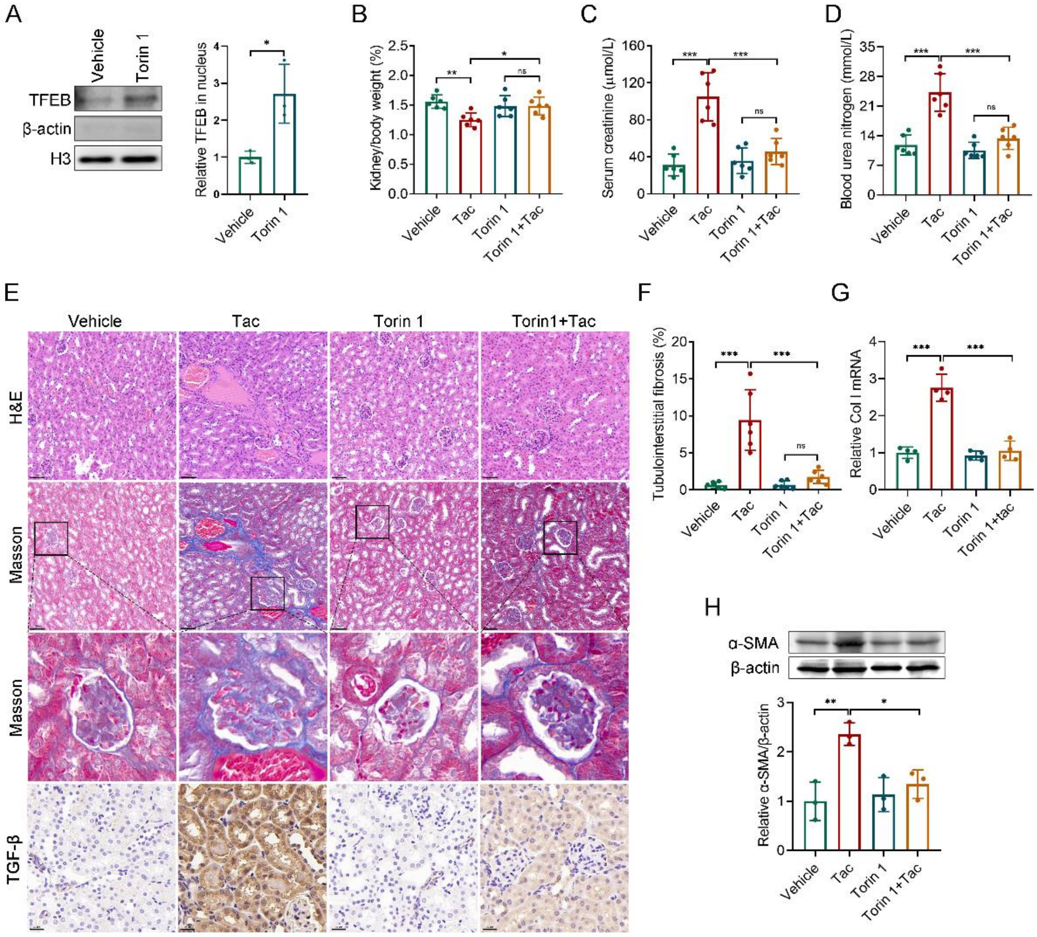

Supplemental Figure 2. TICN was alleviated by the activation of TFEB by torin 1 in vivo. (**A**) TFEB nuclear translocation in kidney tissues of mice treated with vehicle or Torin 1 was evaluated by western blot (n=3). (**B**) The kidney/body weight ratio of mice was recorded (n=6). The levels of (**C**) serum creatinine and (**D**) blood urea nitrogen in mice were determined with commercial kits (n=6). (**E**) Representative images of H&E- and Masson-stained kidney sections (scale bar, 50 μm), as well as TGF-β immunohistochemical staining (scale bar, 20 μm). (**F**) The proportion of tubulointerstitial fibrosis in mouse renal tissue stained by Masson was quantified (n=6). (**G**) *Col1a1* mRNA and (**H**) α-SMA protein in mouse renal tissue was measured by qPCR and western blot, respectively (n=3). Data are shown as mean ± SD and analyzed by two-tailed Student's t-tests (A) and one-way ANOVA (B-D and F-H). *P < 0.05, **P < 0.01, ***P < 0.001.

Supplemental Table. The overlapping genes of TFEB direct target genes identified in human embryonic kidney 293 cells[23] and differentially expressed genes in the kidneys of wild-type or kidney-specific TFEB overexpressed mice[24]

Actr1a	*Gadd45g*	*Pim1*	*Soat1*
Atf5	*Hsd11b2*	*Rabgef1*	*Specc1*
Cdc25b	*Il11*	*Rps6ka1*	*Tbc1d14*
Cln3	*Itfg2*	*Scamp5*	*Tbx3*
Cyp2s1	*Map3k1*	*Sfxn3*	*Tmprss2*
Eral1	*Mlph*	*Slc20a1*	*Trib3*
Gadd45a	*Pdgfa*	*Slc38a1*	*Wdfy1*

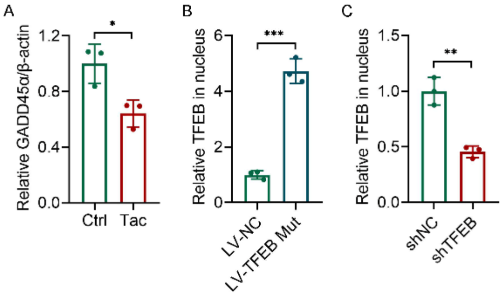

Supplemental Figure 3. (**A**) GADD45α expression levels in mice treated with vehicle or tacrolimus were quantified (n=3). (**B**) TFEB expression levels in HK-2 cells infected with LV-NC or LV-*TFEB* Mut were normalized to H3 (n=3). (**C**) TFEB expression levels in HK-2 cells infected with shNC or sh*TFEB* were normalized to H3 (n=3). Data are shown as mean ± SD and analyzed by two-tailed Student's t-tests. *P < 0.05, **P < 0.01, ***P < 0.001.

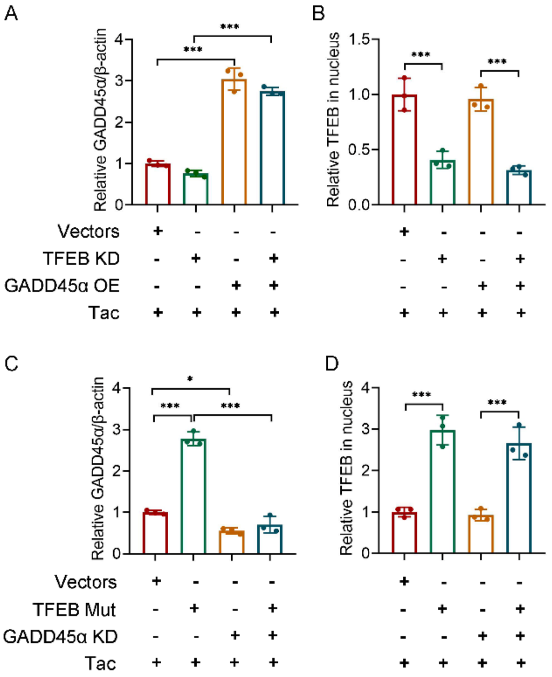

Supplemental Figure 4. Densitometric analysis of (**A**) TFEB and (**B**) GADD45α blots in HK-2 cells transfected with control vectors, *TFEB* shRNA, LV-*GADD45A* or combination of the latter two and then treatd with Tac (50 μM for 24 h) (n=3). Densitometric analysis of (**C**) TFEB and (**D**) GADD45α blots in HK-2 cells transfected with control vectors, LV-*TFEB* Mut, sh*GADD45A* or combination of the latter two and then treatd with Tac (50 μM for 24 h) (n=3). Data are shown as mean ± SD and analyzed by one-way ANOVA. *P < 0.05, **P < 0.01, ***P < 0.001.
