# Supplementary figures and images for: GADD45α is a direct target of TFEB and contributes to tacrolimus-induced chronic nephrotoxicity
Source: JCI Insight. 2025 Feb 6;10(6):e183560. doi: 10.1172/jci.insight.183560 (PMC11949043; doi:10.1172/jci.insight.183560)

Figure 1

1B

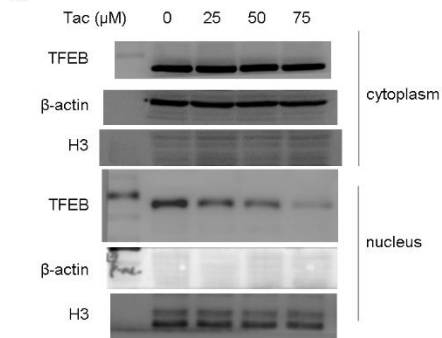

1F

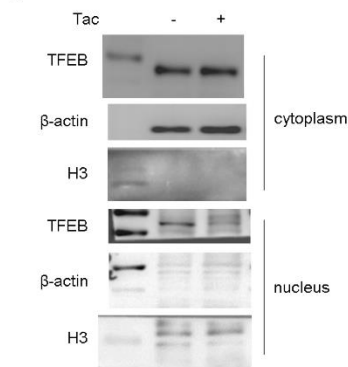

Figure 2

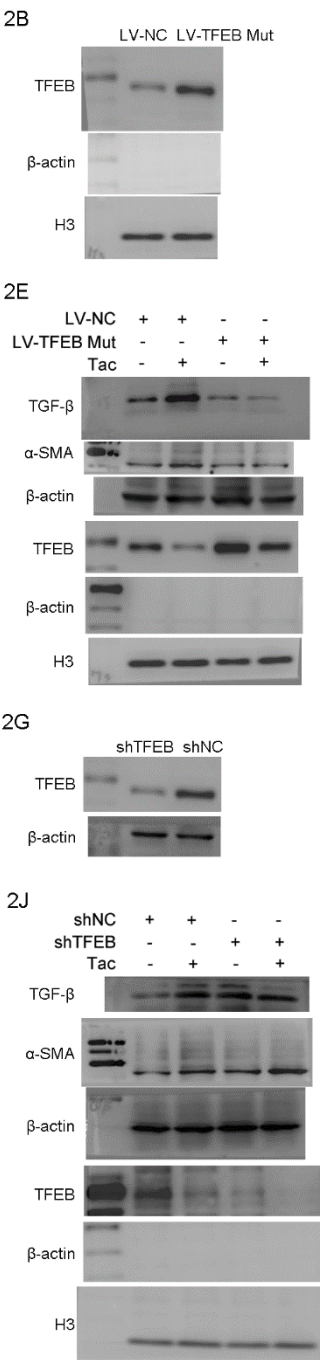

Figure 3

3A

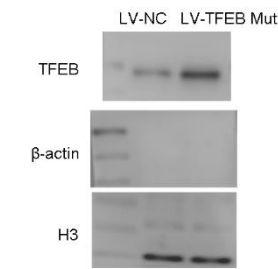

3H

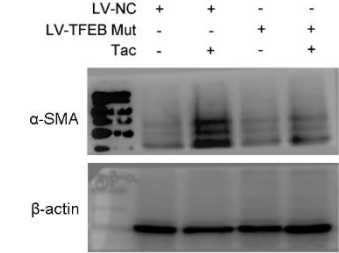

Supplementary figure 2

S2A

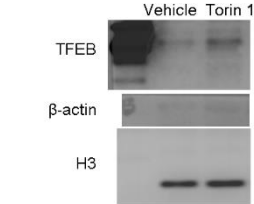

S2H

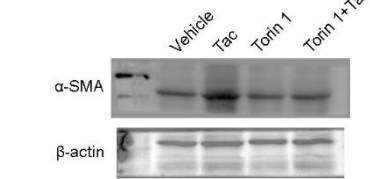

Figure 4

4C

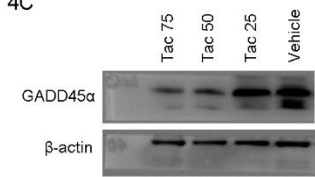

4E

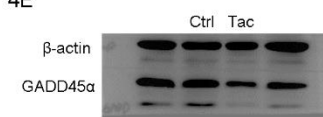

4K

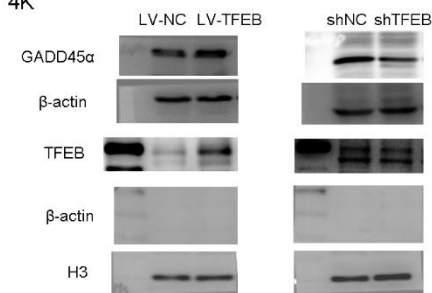

Figure 5

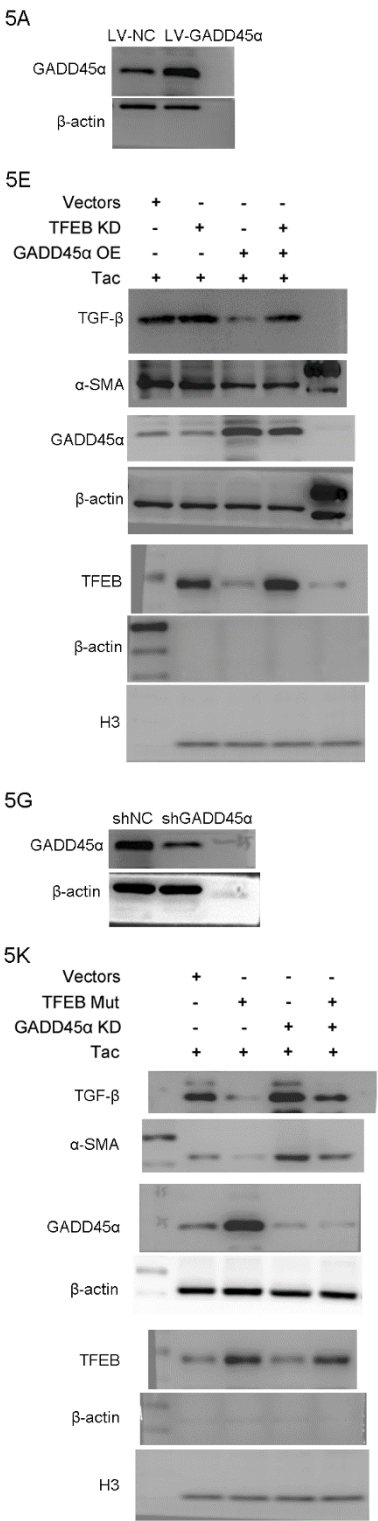

Figure 6

6A

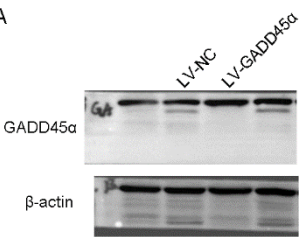

6H

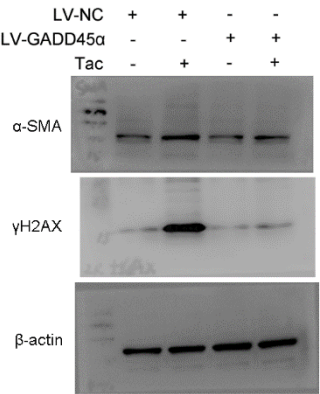

Supplement: Unedited blot and gel images [file jciinsight-10-183560-s045.pdf]
